# Acquired agitation in acute respiratory distress syndrome with COVID-19 compared to influenza patients: a propensity score matching observational study

**DOI:** 10.1186/s12985-022-01868-1

**Published:** 2022-09-10

**Authors:** Adel Maamar, Clémence Liard, Willelm Doucet, Florian Reizine, Benoit Painvin, Flora Delamaire, Valentin Coirier, Quentin Quelven, Pauline Guillot, Mathieu Lesouhaitier, Jean Marc Tadié, Arnaud Gacouin

**Affiliations:** 1 CHU Rennes, Service de Maladies Infectieuses et Réanimation Médicale, Hôpital Pontchaillou, Université de Rennes 1, 2, rue Henri Le Guilloux, 35033 Rennes Cedex 9, France; 2 Faculté de Médecine, Université de Rennes 1, Unité INSERM CIC 1414, IFR 140, Rennes, France

**Keywords:** Covid-19, Influenza, ARDS, ICU, Agitation, Encephalopathy

## Abstract

**Background:**

A growing body of evidence reports that agitation and encephalopathy are frequent in critically ill Covid-19 patients. We aimed to assess agitation’s incidence and risk factors in critically ill ARDS patients with Covid-19. For that purpose, we compared SARS-CoV-2 acute respiratory distress syndrome (ARDS) patients with a population of influenza ARDS patients, given that the influenza virus is also known for its neurotropism and ability to induce encephalopathy.

**Methods:**

We included all the patients with laboratory-confirmed Covid-19 infection and ARDS admitted to our medical intensive care unit (ICU) between March 10th, 2020 and April 16th, 2021, and all the patients with laboratory-confirmed influenza infection and ARDS admitted to our ICU between April 10th, 2006 and February 8th, 2020. Clinical and biological data were prospectively collected and retrospectively analyzed. We also recorded previously known factors associated with agitation (ICU length of stay, length of invasive ventilation, SOFA score and SAPS II at admission, sedative and opioids consumption, time to defecation). Agitation was defined as a day with Richmond Agitation Sedation Scale greater than 0 after exclusion of other causes of delirium and pain. We compared the prevalence of agitation among Covid-19 patients during their ICU stay and in those with influenza patients.

**Results:**

We included 241 patients (median age 62 years [53–70], 158 males (65.5%)), including 146 patients with Covid-19 and 95 patients with Influenza. One hundred eleven (46.1%) patients had agitation during their ICU stay. Patients with Covid-19 had significantly more agitation than patients with influenza (respectively 80 patients (54.8%) and 31 patients (32.6%), *p* < 0.01). After matching with a propensity score, Covid-19 patients remained more agitated than influenza patients (49 (51.6% vs 32 (33.7%), *p* = 0.006). Agitation remained independently associated with mortality after adjustment for other factors (HR = 1.85, 95% CI 1.37–2.49, *p* < 0.001).

**Conclusion:**

Agitation in ARDS Covid-19 patients was more frequent than in ARDS influenza patients and was not associated with common risk factors, such as severity of illness or sedation. Systemic hyperinflammation might be responsible for these neurological manifestations, but there is no specific management to our knowledge.

**Supplementary Information:**

The online version contains supplementary material available at 10.1186/s12985-022-01868-1.

## Introduction

It is clear that neurological manifestations such as headache, dizziness, confusion, hypogeusia, hyposmia, are common in SARS-CoV-2 patients, and more frequent in critically ill patients [1] although consciousness impairment before intensive care unit (ICU) admission remains rare [2].

Interestingly, it has recently been reported that critically ill patients with Covid-19 frequently experienced encephalopathy with agitation and confusion during the ICU stay [2, 3]. In a cohort study, Helms et al. reported an incidence of 69.3% of patients presenting an unexpected state of agitation [3]. Noteworthy, brain magnetic resonance imaging showed in some of these patients a leptomeningeal enhancement and cerebral blood flow abnormalities as well as ischemic stroke [1], and cerebrospinal fluid analysis revealed inflammatory disturbances [3, 4]. However, these anomalies are very low compared to the incidence of agitation. SARS-CoV-2, like others coronaviruses, invades cells by using the receptor angiotensin-converting enzyme 2 (ACE2) [5, 6]. This receptor is expressed by ciliated upper respiratory cells, neurons and glial cells [7]. Although the mechanisms involved remain incompletely understood, this neurotropism might explain the neurologic symptoms, such as agitation in ICU.

On the other hand, acquired encephalopathy in ICU is frequent [8], and is associated with sedation, age, the severity of illness [9], ileus [10], and cardiovascular risk factors [11]. Moreover, it has been previously described that agitation, after exclusion of other causes of delirium, is well correlated with delirium and encephalopathy [12]. The issue is important as agitated patients in ICU have a higher risk of increased length of stay, medical complications, higher costs, and poor outcomes including increased mortality [13].

Many questions remain unclear, especially if critically ill patients with Covid-19 who require invasive ventilation are more agitated than other critically ill patients, especially patients infected with other respiratory viruses.

Although some studies assessed the prevalence of delirium or encephalopathy in critically ill Covid-19 patients, there was no comparator group. SARS-CoV-2 and influenza virus are both respiratory viruses and share specific features, especially acute respiratory distress syndrome (ARDS), the observation of bilateral pulmonary infiltrates, the possible corticosteroid administration, and the systemic dysregulation of immune functions. Furthermore, the influenza virus is the most important pathogen responsible of acute encephalopathy [14], especially in children. Although the pathophysiology is not well understood, several underlying mechanisms have been proposed, with an important overlap such as influenza-induced metabolic disorders, encephalopathy associated with hypercytokinemia and vasogenic cerebral edema, and encephalopathy with localized cortical edema [15]. Several reports found an abnormally high level of inflammatory cytokines, such as tumor necrosis factor and interleukin-6, in serum and cerebrospinal fluid during the acute stage of influenza encephalopathy [16].

For that purpose, we compared the incidence of agitation in SARS-CoV-2 ARDS patients and in influenza ARDS patients, and compared the demographic, clinical and biologic features between these patients.

## Methods

### Study design and population

We conducted a monocentric prospective observational study in the medical intensive care unit of Rennes University Hospital, a tertiary teaching hospital.

Data were recorded prospectively and retrospectively analyzed. We included all consecutive patients over 18 years with laboratory-confirmed Covid-19 infection and mechanically ventilated (MV) who were admitted to the ICU between March 10th, 2020 to April 16th, 2021 for patients with Covid-19, and between April 10th, 2006 to February 8th, 2020 for patients with influenza. Notably, patients were tested for both influenza and SARS-Cov-2 during the Covid-19 pandemic period.

Only laboratory-confirmed cases were included. A confirmed case of Covid-19 or influenza was defined by a positive result on a reverse-transcriptase–polymerase-chain-reaction (RT-PCR) using the Influenza A/B r-geneTM (Argene®, bioMérieux, Marcy-l’Etoile, France) and TaqPath™ Covid-19 (Thermo Fisher Scientific, Illkirch-Grafenstaden, France) assay of a specimen collected on an endotracheal aspiration. ARDS definition was based on Berlin criteria [17]. This study was conducted in accordance with the Declaration of Helsinki and approved by the hospital’s ethical committee (No. 20.52) and we followed the Strengthening the Reporting of Observational Studies in Epidemiology (STROBE) recommendations for cohort studies. Due to its observational nature, patient signed informed consent was waived by the ethical committee in compliance with French legislation on observational studies of anonymized data.

### Patients management

Sedation and pain management are standardized in our ICU according to recommended guidelines to decrease the risk of oversedation [18]. Thus, sedation and analgesia were managed through a nurse-driven protocol with assessment of the Richmond Agitation Sedation Scale [19] (RASS) and the Behavioral Pain Scale [20] (BPS) or Numeric Rating Scale (NRS) in those able to communicate. Sedation was primarily targeted at a light level (RASS between 0 and − 1), except for ARDS patients, for whom sedation was targeted at a deep level (RASS − 5) before introducing neuromuscular blocking agents, according to the ACURASYS sedation protocol [21]. After improvement of the ARDS, sedation was reduced to target a light level (RASS between 0 and − 1). Analgesia was targeted to obtain a BPS < 5 or NRS < 3. There was no daily sedation interruption. Sedatives used were midazolam or propofol, and opioid used was morphine.

All ARDS patients in both groups received protective ventilation according to published guidelines [22], especially: assist-control mode, initial tidal volume targeted at 6 mL per kilogram of predicted body weight, titration of positive end-expiratory pressure (PEEP) level and end-inspiratory pressure measured at least every 2 h to be kept below 28 cm of water.

### Data collection

We collected demographic data, clinical symptoms at presentation, and comorbidities (hypertension, obesity, diabetes mellitus, and neurologic history). Diabetes mellitus was defined by a history of diabetes requiring chronic therapy with insulin or an oral hypoglycemic agent. Obesity was defined by a body mass index greater than or equal to 30 kg per square of height in meters [23]. The severity of illness was assessed through severity scores at admission to ICU, including Simplified Acute Physiology Score [24] (SAPS) II within 24 h after admission and Sequential Organ Failure Assessment [25] (SOFA) score calculated on the first day after admission. As previously described, we defined agitation as a day with RASS score greater than 0 during the ICU stay [26] not explained by pain (i.e. BPS < 3) and other causes of delirium (alcoholic, iatrogenic, or metabolic). We also recorded parameters that can contribute to encephalopathy or agitation in critically ill patients: the cumulative dose of midazolam, propofol, and opioids during the ICU stay, length of sedation, and time to defecation defined as the delay between admission in the ICU and the first defecation [10]. Moreover, we collected events that may result from agitation, such as the need for anti-agitation drugs, and the self-extubation, defined as a deliberate action taken by the patient to remove the endotracheal tube. Finally, patients were followed from admission to day 28, and we recorded the 28 days of survival.

### Statistical analysis

Normally distributed continuous variables are presented as the means ± standard deviations (SDs), whereas non-normally distributed data are presented as medians (interquartile ranges (IQRs)). Categorical variables are presented as numbers (percentages). Continuous variables were compared by using the Mann–Whitney U test. Proportions for categorical variables were compared using the χ^2^ test or Fisher’s exact test when more appropriate.

To improve the balance of baseline characteristics and reduce the effects of selection bias and potential confounding factors in this observational study, a propensity score analysis was performed. The propensity score was calculated by a multivariable logistic regression model using a priori defined variables with a clinical relevance between the two groups (age, BMI, MV and sedation duration, ileus duration, cumulative doses of midazolam, propofol, and morphine, day-28 survival, Glasgow coma scale at admission). These variables were chosen based on the results of previous studies [9, 10, 18]. The Covid-19 and influenza patients were then matched 1:1 on these propensity scores.

Second, we used a Cox-proportional hazard model to determine whether agitation during the ICU stay was independently associated with mortality on day 28. For this analysis, susceptibilities known to produce agitation achieving a *p* value of 0.10 were used for adjustments. Results were expressed as Hazard Ratios (HR) with their 95% confident interval (CI). Survival curves were constructed until day 28 using the Kaplan–Meier method and compared with the log-rank test. Patients alive on day 28 were censored. We defined agitation-free days as the number of days in the first 28 days after admission during which the patient was alive without agitation and not in a coma for any cause. Patients who died within the 28-day study period were recorded as having zero days free of agitation. As agitation is associated with mortality, death was deemed as a competing risk for agitation in a multivariate analysis using a Fine-gray model to fit cumulative incidence. All probability values reported were 2-sided. Statistical analyses were performed using R 4.1.2 (R Foundation for Statistical Computing, Vienna, Austria), and p values of less than 0.05 were considered significant.

## Results

### Baseline characteristics of the patients

**Fig. 1:**
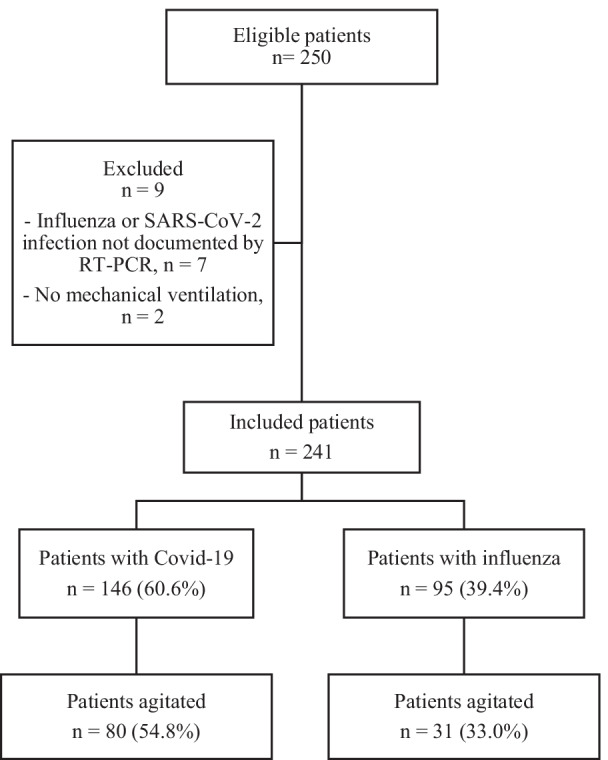
Diagram flow chart

We included 241 patients: 146 Covid-19 patients (60.6%) and 95 Influenza patients (39.4%) (Fig. 1). Of note, we found no co-infection with influenza and SARS-CoV-2 since the beginning of the Covid-19 pandemics.

### Comparing Covid-19 with Influenza patients

#### Unmatched population

The characteristics of the 241 patients and their comparison are summarized in Table 1. The median age was 62 [53–70] years, and the median SAPS II was 37 [28–49] points. The median length of stay in ICU was 15 [10–28] days, and the survival rate on day 28 was 87.1%. One hundred eleven (46.2%) patients experienced agitation during their ICU stay. Compared to patients with influenza, patients with Covid-19 had more hypertension (48.6% vs. 25.2%, *p* < 0.001), more diabetes (24.7% vs. 12.6%, *p* = 0.03), more obesity (72.6% vs. 23.2%, *p* < 0.001) and their level of consciousness assessed by the Glasgow coma scale at admission was significantly higher (15 [15] vs. 15 [14, 15], *p* = 0.006). There was no difference in neurological medical history. At admission, patients with Covid-19 were less severe than patients with influenza (SAPS II score 33 [24–42] vs. 46 [35–60], *p* < 0.001). There was no significant difference in the cumulative doses of sedation between the 2 groups, except for the propofol (6938 mg [1694–16375] vs. 3400 mg [1050–7550], *p* = 0.011). Of note, the median length of ileus was not significantly different between the two groups (7 days [5–10] vs. 6 days [4–9], *p* = 0.075). The median length of MV and stay in ICU were not significantly different (respectively 13 days [8–21] vs. 14 days [9–24], *p* = 0.238, and 15 days [10–26] vs. 17 days [9–28], *p* = 0.941).

**Table 1:** Baseline characteristics and outcomes

	Overall (n = 241)	Covid-19 (n = 146)	Influenza (n = 95)	*p* value
Age—years	62 [53–70]	65 [55–72]	59 [51–65]	< 0.001
Male	158 (65.5)	99 (67.8)	59 (62.1)	0.44
Medical history				
Hypertension	95 (39.4)	71 (48.6)	24 (25.2)	< 0.001
Diabetes mellitus	48 (19.9)	36 (24.7)	12 (12.6)	0.03
Obesity	128 (53.1)	106 (72.6)	22 (23.2)	< 0.01
Neurological medical history	35 (14.5)	18 (12.3)	17 (17.9)	0.31
BMI—kg/m^2^	28.2 [24.6–32.0]	28.7 [25.9–32.9]	27.0 [23.8–31.2]	0.05
GCS at admission	15 [15–15]	15 [15–15]	15 [14, 15]	0.001
Severity during the first 24 h				
SAPS II	37 [28–49]	33 [24–42]	46 [35–60]	< 0.001
SOFA	6 [4–8]	4 [3–7]	9 [7–11]	< 0.001
Worst PaO_2_/FiO_2_—mmHg	97.5 [74.0–131.8]	105.5 [83.0–142.3]	84.0 [63.3–114.8]	< 0.001
Agitation	111 (46.2)	80 (54.8)	31 (33.0)	< 0.001
Administration of anti-agitative drug	100 (42.6)	76 (53.9)	24 (25.5)	< 0.001
Sedation				
Length of sedation—days	10 [6–17]	11 [6–17]	10 [5–15]	0.12
Cumulative doses of morphine—mg	987 [521–2345]	975 [530–2187]	1200 [434–2868]	0.81
Cumulative doses of midazolam—mg	980 [503–2326]	966 [503–2164]	1162 [514–2753]	0.70
Cumulative doses of propofol—mg	13,135 [1426–13607]	6938 [1694–16375]	3400 [1050–7550]	0.01
Length of MV—days	13 [8–23]	13 [8–21]	14 [9–24]	0.24
Length of stay in ICU—days	15 [10–28]	15 [10–26]	17 [9–28]	0.94
Length of ileus—days	6 [4–9]	7 [5–10]	6 [4–9]	0.07
Self-extubation	21 (8.8)	13 (9.0)	8 (8.4)	1.00
28-days survival	210 (87.1)	133 (91.1)	77 (81.1)	0.04

Data are presented as number (%) or median [interquartile range]

*BMI* body mass index, *GCS* Glasgow coma scale, *SAPS* simplified acute physiology score, *SOFA* sequential organ failure assessment, *MV* mechanical ventilation, *ICU* intensive care unit

Finally, patients with Covid-19 experienced significantly more agitation than patients with influenza (80 patients (54.8%) vs. 31 patients (33.0%), *p* < 0.001).

#### Matched population

Table 2 compares Covid-19 and influenza-matched patients after performing a propensity score analysis. After matching, agitation was significantly higher in Covid-19 patients than influenza patients (52 (54.7%) vs. 32 (33.7%), *p* = 0.006), although there was no significant difference concerning cumulative doses of midazolam, propofol, or morphine, ICU length of stay, duration of mechanical ventilation and Glasgow coma scale at admission.

**Table 2:** Baseline characteristics and outcomes in the matched population

	Covid-19 (n = 95)	Influenza (n = 95)	*p* value
Age—years	59 [50–68.5]	59 [50.5–65]	0.42
Male	66 (69.5)	59 (62.1)	0.36
Medical history			
Hypertension	42 (44.2)	24 (25.3)	0.01
Diabetes mellitus	21 (22.1)	12 (12.6)	0.13
Obesity	68 (71.6)	22 (23.2)	< 0.001
BMI—kg/m^2^	28.6 [26–33.6]	27 [23.8–31.1]	0.06
GCS at admission	15 [15–15]	15 [15–15]	0.12
Severity during the first 24 h			
SAPS II	32 [22–42]	46 [35–60]	< 0.001
SOFA	4 [3–7]	9 [7–11]	< 0.001
Worst PaO_2_/FiO_2_	107 [83–147]	83 [63–114.5]	< 0.001
Agitation	52 (54.7)	32 (33.7)	0.006
Sedation			
Length of sedation—days	9 [6–15]	10 [5–15]	0.53
Cumulative doses of morphine—mg	922 [540–1601]	1148 [414–2848]	0.47
Cumulative doses of midazolam—mg	879 [451.5–1506.5]	1136 [452–2652.5]	0.28
Cumulative doses of propofol—mg	6060 [2639–14080]	3930 [1120–11790]	0.06
Length of MV—days	13 [7–20]	14 [9–24]	0.13
Length of stay in ICU—days	13 [9–24]	17 [9–28]	0.4
Length of ileus—days	7 [4–9]	6 [4–8.5]	0.41
Self-extubation	13 (13.7)	8 (8.4)	0.36
28-days survival	83 (87.4)	77 (81.1)	0.32

Data are presented as number (%) or median [interquartile range]

*BMI* body mass index, *GCS* Glasgow coma scale, *SAPS* simplified acute physiology score, *SOFA* sequential organ failure assessment, *MV* mechanical ventilation, *ICU* intensive care unit

#### Mortality on day 28 from admission to the ICU and impact of agitation

Survival at day-28 of the full population was significantly higher in the Covid-19 group than the influenza group (133 (91.1%) vs. 77 (81.1%), *p* = 0.038) (Table 1). Agitation remained independently associated with mortality after adjustment for other factors (HR = 1.85, 95% CI 1.37–2.49, *p* < 0.001) (Table 3). Of note, duration of MV (HR = 1.07 by 1-day increment, 95% CI 1.05–1.08, *p* < 0.001) and duration of ileus (HR = 1.14 by 1-day increment, 95% CI 1.1–1.18, *p* < 0.001) were also independently associated with mortality.

**Table 3:** Multivariable analysis of factors associated with 28-days mortality

Variables	HR	95% CI	*p* value
Agitation	1.85	[1.37–2.49]	< 0.001
Male sex	0.87	[0.65–1.16]	0.34
Length of sedation	0.99	[0.98–1.01]	0.21
Length of MV	1.07	[1.05–1.08]	< 0.001
Length of ileus	1.14	[1.10–1.18]	< 0.001
Cumulative dose of propofol	1.00	[1.00–1.00]	0.32
SAPS II	0.99	[0.99–1.00]	0.11

*HR* Hazard ratio, *CI* confidence interval, *MV* mechanical ventilation, *SAPS* simplified acute physiology score

**Fig. 2:**
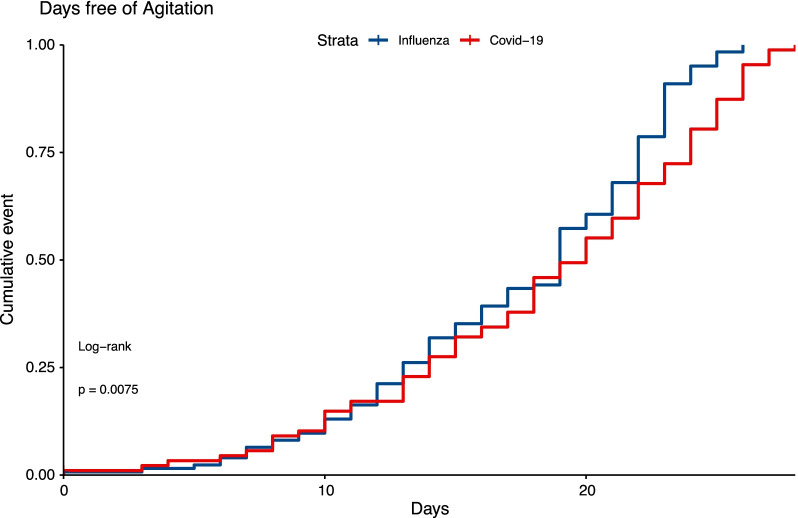
Cumulative incidence for agitation-free days in Covid-19 and influenza patients

Moreover, Covid-19 patients had a lower probability of agitation free-days during their ICU stay (p = 0.008 by log-rank test, Fig. 2), and the cumulative incidence of agitation free-days was lower in Covid-19 patients according to the competing risk analysis (Additional file 1: Fig. S1).

## Discussion

In this single-center study, agitation in ARDS Covid-19 patients was frequent (54.8%) during their ICU stay and was significantly more frequent than in ARDS influenza patients (33.0%). After propensity score matching, agitation remained significantly higher in Covid-19 patients than in influenza patients, while other known risk factors as cumulative doses of sedatives and opioids, ICU length of stay, duration of mechanical ventilation, duration of sedation, or duration of ileus, did not differ. Moreover, we found that this agitation was independently associated with an increased mortality. Our findings are consistent with other studies that found that delirium is frequent in Covid-19 patients although the prevalence of delirium or encephalopathy was ranging from 15 to 84% [3, 27, 28], probably due to different definitions and assessment methods of delirium. For instance, Mao et al. also reported that neurologic symptoms were more common in patients with severe Covid-19 infection [2]. Interestingly, although our study population was systematically tested for both viruses, no co-infection has been found in our cohort. This is consistent with the results of other studies assessing the incidence of SARS-CoV-2 and Influenza co-infections which was very low, ranging from zero to 0.3% [29, 30].

To our knowledge, this study is the only one to assess critically ill ARDS patients with Covid-19 for agitation compared to critically ill ARDS patients infected by another respiratory virus. We chose the influenza virus as comparator because both influenza and SARS-CoV-2 are respiratory viruses with known neurotropism and the ability to induce encephalopathy [3, 14]. Furthermore, they allowed us to compare two homogeneous populations of ARDS patients exposed to the same risk factors, especially with similar lengths of mechanical ventilation and ICU stay, even in the unmatched cohort.

Contrary to previous studies, we did not find in our cohort Covid-19 patients more heavily sedated by benzodiazepines [18], which is generally considered a risk factor for delirium and coma in the ICU setting [31]. These findings suggest that agitation and encephalopathy in critically ill Covid-19 patients are probably specific to SARS-CoV-2 infection and cannot be attributed to previously described factors associated with encephalopathy in ICU [32], such as sedation [8] or ileus [10].

Indeed, SARS‐CoV‐2, like others coronaviruses, has a spike protein that has an avid affinity for ACE2 on human cells [6]. SARS-CoV-2 can reach the central nervous system (CNS) by four routes: (A) the hematopoietic pathway and subsequent rupture of the blood–brain barrier (BBB); (B) via blood-cerebrospinal fluid (CSF); (C) transsynaptic viral spreading; (D) through the entry to circumventricular organs (CVO) [33]. SARS-CoV-2 interactions with ACE2 could cause astrogliosis and microgliosis, increase BBB permeability, allow monocyte and leukocyte infiltration to the CNS, and lead to nerve cells dysfunction [34].

In addition to a direct central nervous system invasion, there is also indirect and non-specific damage, such as release of inflammatory mediators and secondary effect of other organ system failure [33]. As described by Helms [3] and Garg [35], CSF analysis showed no direct virus detection in the samples but inflammatory disturbances with pleocytosis and raised proteins. It is consistent with magnetic resonance imaging results showing subarachnoid contrast enhancement suggestive of abnormal permeability of the blood meningeal barrier or microbleeds [3, 35], supporting that the brain damages are, in most cases, probably related to inflammatory or immune-mediated response rather than a direct infiltration of the central nervous system.

The state of agitation may be part of a picture of Covid-19 encephalopathy, a rapidly developing pathobiological process in the brain that can lead to a clinical presentation of delirium, or in case of a severely decreased level of consciousness, coma; all representing a change from baseline cognitive status [36]. Such encephalopathy can have important negative implications for Covid-19 patients since acute brain dysfunction in ICU is associated with long-term dementia and post-intensive care syndrome [37–39]. Although agitation was not associated with increased mortality, treatment or prevention of agitation of Covid-19 patients in ICU seems mandatory. However, data are lacking for the management of such cases. Kotfis et al. suggest a bundle for management of Covid-19 delirium [32], but cannot recommend any specific care, whether pharmacological or not.

Some limitations should be acknowledged. First, in our ICU we do not use in routine dedicated tools, such as Confusion Assessment Method-ICU [40] (CAM-ICU). However, although the routine use of CAM-ICU is recommended in clinical practice guidelines, the adherence to the guidelines is still low [41]. Thus, agitation based on the RASS appears to be a “real-life” clinical symptom, routinely assessed and intuitive, especially during the actual pandemic and the resulting burden generated in critical care units. Additionally, several studies have concluded that a RASS score > 0 after exclusion of other causes of delirium (for instance, pain, iatrogenic, metabolic, or alcoholic) is well correlated with delirium and/or encephalopathy [12]. For these reasons, we retained this definition. Second, after exclusion of other causes, agitation might be a clinical sign of hyperactive delirium, and we cannot estimate hypoactive delirium in our study, which impacts patients outcome [42]. Nevertheless, hyperactive delirium appears to be the most common delirium encountered in critically ill Covid-19 patients and a recent study found that hyperactive delirium accounted for 86.6% of the patients [3]. Lastly, due to several restrictions in family visits during the Covid-19 pandemic could have significantly impacted the occurrence of delirium in patients, even with very similar clinical management between Covid-19 and Influenza patients in our ICU. Of note, it was shown that family presence is associated with a reduction of delirium in severe Covid-19 patients [28].

## Conclusion

Our results showed SARS-CoV-2 is more frequently associated with agitation in ARDS patients than influenza. The agitation was not associated with common risk factors, such as the severity of illness or sedation. These findings suggest that SARS-CoV-2 is, directly and indirectly involved in agitation and should probably be acknowledged as a risk factor. As agitation can be one of the presentations of a delirium, its presence should alert us to the risk of encephalopathy.

## Supplementary Information

**Additional file 1.** Cumulative incidence of competing risk for agitation-free days in Covid-19 and influenza patients.

## Data Availability

The data that support the findings of this study are available from the corresponding author, AM, upon reasonable request.
